# Impact of Face-to-Face Teaching in Addition to Electronic Learning on Personal Protective Equipment Doffing Proficiency in Student Paramedics: Protocol for a Randomized Controlled Trial

**DOI:** 10.2196/26927

**Published:** 2021-04-30

**Authors:** Loric Stuby, Ludivine Currat, Birgit Gartner, Mathieu Mayoraz, Stephan Harbarth, Laurent Suppan, Mélanie Suppan

**Affiliations:** 1 Emergency Medical Services Genève TEAM Ambulances Geneva Switzerland; 2 ESAMB - École Supérieure de Soins Ambulanciers College of Higher Education in Ambulance Care Geneva Switzerland; 3 Division of Emergency Medicine Department of Anesthesiology, Clinical Pharmacology, Intensive Care and Emergency Medicine University of Geneva Hospitals and Faculty of Medicine Geneva Switzerland; 4 MEDI - Center for Medical Education College of Higher Education in Ambulance Care Bern Switzerland; 5 Infection Control Program and WHO Collaborating Centre on Patient Safety University of Geneva Hospitals and Faculty of Medicine Geneva Switzerland; 6 Division of Anesthesiology Department of Anesthesiology, Clinical Pharmacology, Intensive Care and Emergency Medicine University of Geneva Hospitals and Faculty of Medicine Geneva Switzerland

**Keywords:** personal protective equipment, electronic learning, prehospital, student paramedics, infection prevention, face-to-face learning, protection, student, online learning, online education, protocol, randomized controlled trial, gamification

## Abstract

**Background:**

The COVID-19 pandemic has brought attention to the importance of correctly using personal protective equipment (PPE). Doffing is a critical phase that increases the risk of contamination of health care workers. Although a gamified electronic learning (e-learning) module has been shown to increase the adequate choice of PPE among prehospital personnel, it failed to enhance knowledge regarding donning and doffing sequences. Adding other training modalities such as face-to-face training to these e-learning tools is therefore necessary to increase prehospital staff proficiency and thus help reduce the risk of contamination.

**Objective:**

The aim of this study is to assess the impact of the Peyton 4-step approach in addition to a gamified e-learning module for teaching the PPE doffing sequence to first-year paramedic students.

**Methods:**

Participants will first follow a gamified e-learning module before being randomized into one of two groups. In the control group, participants will be asked to perform a PPE doffing sequence, which will be video-recorded to allow for subsequent assessment. In the experimental group, participants will first undergo face-to-face training performed by third-year students using the Peyton 4-step approach before performing the doffing sequence themselves, which will also be video-recorded. All participants will then be asked to reconstruct the doffing sequence on an online platform. The recorded sequences will be assessed independently by two investigators: a prehospital emergency medicine expert and an infection prevention and control specialist. The assessors will be blinded to group allocation. Four to eight weeks after this first intervention, all participants will be asked to record the doffing sequence once again for a subsequent skill retention assessment and to reconstruct the sequence on the same online platform to assess knowledge retention. Finally, participants belonging to the control group will follow face-to-face training.

**Results:**

The study protocol has been presented to the regional ethics committee (Req-2020-01340), which issued a declaration of no objection as such projects do not fall within the scope of the Swiss federal law on human research. Study sessions were performed in January and February 2021 in Geneva, and will be performed in April and June 2021 in Bern.

**Conclusions:**

This study should help to determine whether face-to-face training using the Peyton 4-step approach improves the application and knowledge retention of a complex procedure when combined with an e-learning module.

**International Registered Report Identifier (IRRID):**

PRR1-10.2196/26927

## Introduction

### Background and Importance

The emergence of COVID-19 has democratized the use of personal protective equipment (PPE) for all health care workers in and outside hospitals [1] in accordance with international recommendations [2]. Donning and doffing procedures contribute to the adequate use of PPE and reduce the risk of self-contamination of caregivers [3]. This last point is critical as frontline health care workers are a scarce and essential resource who are at increased risk of being contaminated [4].

Although the donning phase is associated with a low risk of contamination, doffing is a critical phase and greatly increases the risk of contamination of caregivers [5-9]. One of the strategies that effectively contributes to reducing staff self-contamination is doffing in structured areas dedicated to the removal of PPE [10,11]. However, this strategy is difficult to apply in the prehospital field, where health care workers must often doff PPE on site. It is therefore all the more relevant to train prehospital health care workers in the noncontaminating removal of PPE so that they can perform it adequately under all circumstances*.*

Two previous studies have shown that training prehospital staff in using PPE through a gamified electronic learning (e-learning) module increases the proportion of making an adequate choice of PPE [12,13]. However, this module failed to improve knowledge acquisition of correct doffing sequences. It was therefore concluded that e-learning training alone is insufficient to adequately train prehospital staff in the noncontaminating removal of PPE, and that other training modalities should be considered, either as standalone interventions or in combination with this module. The Peyton 4-step teaching approach [14] is an effective training structure that has been shown to increase knowledge and skill retention when compared to standard training in the acquisition of procedural skills [15]. Our hypothesis is that adding a face-to-face training modality using the Peyton 4-step approach to a gamified e-learning module could increase the proficiency of prehospital workers regarding this procedure, thus reducing the risk of contamination of prehospital staff [16].

### Objectives

The primary aim of this study is to define whether the Peyton 4-step approach used in addition to our gamified e-learning module for teaching noncontaminant PPE removal increases the percentage of correct PPE doffing sequences performed by first-year paramedic students in comparison to e-learning alone.

## Methods

### Study Design and Setting

**Figure 1:**
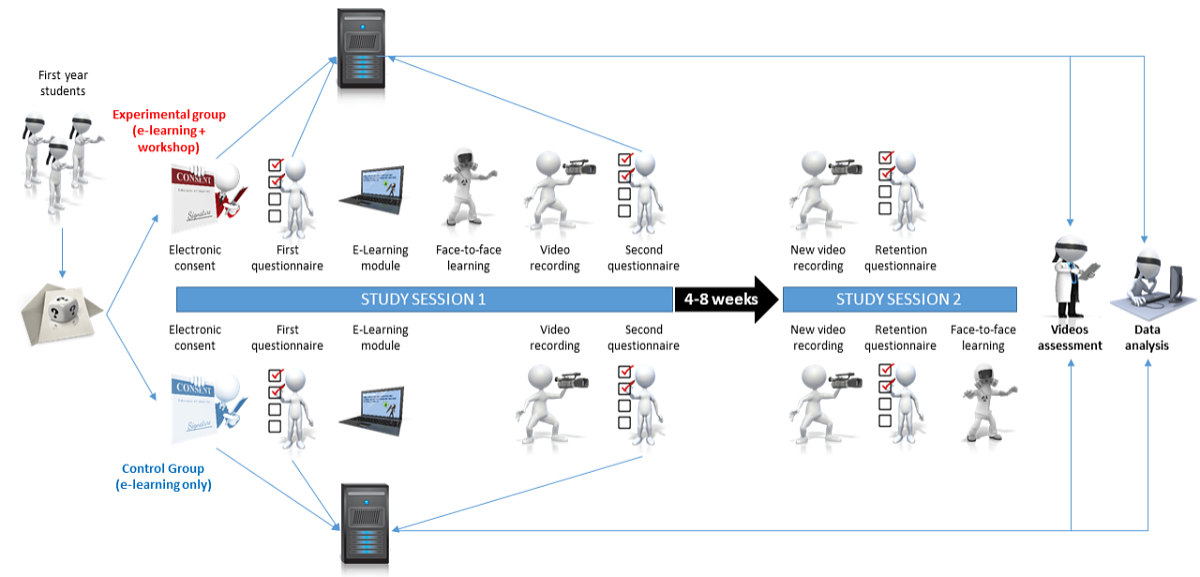
Study design.

We will carry out a parallel-group, randomized, quadruple-blind (participants, instructors, outcome assessors, and data analyst) controlled superiority trial designed following the SPIRIT statement (see Multimedia Appendix 1 for the SPIRIT Checklist) [17], and including relevant elements from the CONSORT-EHEALTH checklist [18] and from the CHERRIES guidelines [19]. The design is detailed in Figure 1.

All first-year students (N=62) from the Colleges of Higher Education in Ambulance Care located in Geneva and Bern, Switzerland, will be invited to take part in this study. To allow the first-year German-speaking students to participate in the study, the study material, including the e-learning module, will be translated into German. Participation will be on a voluntary basis during their school time as part of their curriculum. There will be no exclusion criteria.

The instructors who will be recruited to take part in this study are third-year paramedic students. Their participation will be on a voluntary basis. They will be specifically trained by two investigators in the noncontaminating removal of PPE (LC) and the Peyton teaching approach (L Stuby). These investigators will be accompanied by a bilingual teacher from the Bernese school to help with potential language issues. Peer teaching or near-peer teaching has been shown to have a positive influence on peer learners and to permit a deeper approach to learning [20], with reduced levels of anxiety among participants [21]. The practicability of peer teaching and near-peer teaching has been assessed for different outcomes, including clinical skills [22,23] or respiratory, cardiac, and blood physiology [24]. This form of teaching has recently been used in another study involving medical students [25]. It is therefore reasonable to extrapolate these results to our population of first-year student paramedics. Instructors will be informed that the objective is to teach the noncontaminating doffing of PPE to first-year students during two training sessions. They will not be aware of the study design and will therefore be blinded to the existence of two different training paths, and consequently to the allocation of participants and to the goals of the study. The instructors will receive a detailed PPE doffing procedure validated by infection prevention and control (IPC) experts (Multimedia Appendix 2) and a summary sheet of the Peyton approach steps (Multimedia Appendix 3). The instructor:learner ratio will range from 1:1 to 1:3 as such ratios have been shown to be particularly efficient [15].

### Online Platform

An online platform [26] running under the Joomla! 3.9 content management system (Open Source Matters) will be developed by L Suppan for the purpose of this study. A survey component (Community Surveys Pro 5.5 CoreJoomla) will be installed on the platform. L Suppan will be the only author to have access to the platform’s administration console. There will be no scheduled maintenance or update on the server during the study period. Once created, tested, and validated, the platform will not be altered before the end of the study period.

### Randomization and Concealment of Allocation

An investigator (MS) who does not know the participants and will have no contact with them will randomly assign the participants into two groups according to a computer-generated list [27] with a 1:1 allocation ratio and stratification by school (Geneva, Bern French-speaking, and Bern German-speaking). Opaque, sealed envelopes containing individual login information will be created and given to local investigators. No one else will have access to the coding list. Given the complete lack of risk to the participants, there will be no unblinding procedure, no data monitoring, and no interim analysis. In addition, there is no need to elaborate plans regarding potential adverse events given the type of intervention and the design of this study.

Participants will be divided between the instructors randomly by one of the investigators (L Stuby or LC) using an online team generator [28].

### Enrollment and Consent

**Figure 2:**
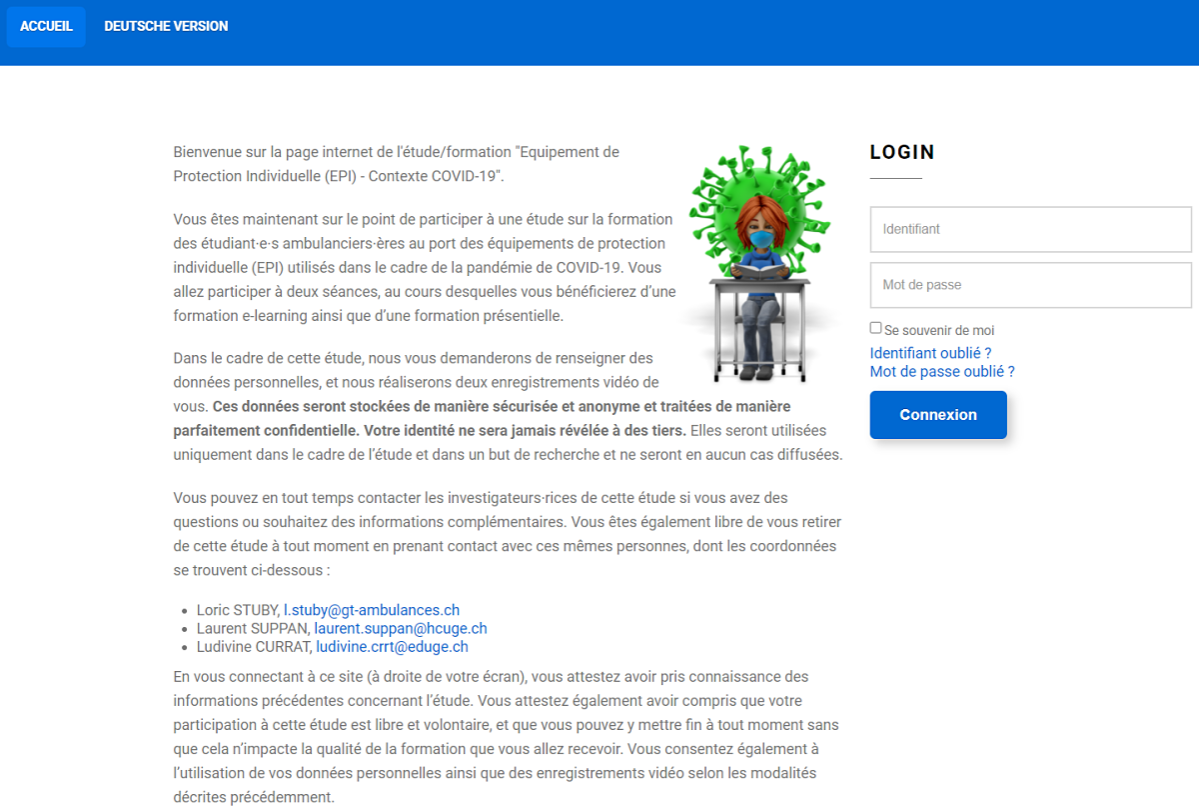
French version of the platform welcome screen.

**Figure 3:**
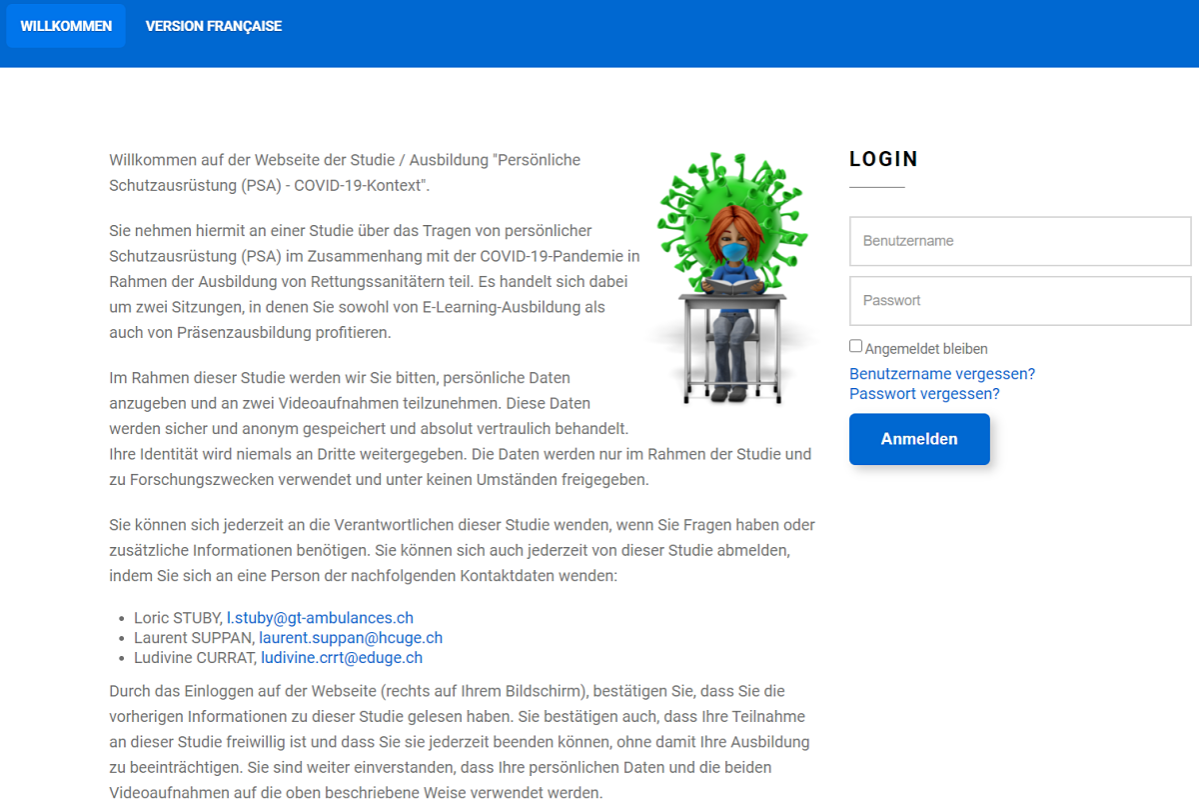
German version of the platform welcome screen.

Prior to the beginning of the study, students will be sent emails containing general information about the study (Multimedia Appendix 4). The home screen of the website will contain information regarding the study, including data security and the learning objectives (Figure 2 and Figure 3). Consent (including for the recording of videos) will be gathered electronically. Participants will be informed that they will attend, during the course of their learning path, a workshop on correct PPE doffing according to good practices validated by IPC experts.

Participation will be free and each participant will be able to withdraw at any time without giving any justification. Participants will benefit from this study by acquiring knowledge regarding the safe doffing of PPE, which will be useful in their practice. There will be no financial compensation or incentive.

The data collected are encoded using the randomly assigned connection identifiers; thus, if a participant keeps their identifiers, it will be possible for them to request that the answers be deleted once the survey is completed.

### Study Sequence

After picking up an envelope, each participant will be asked to log into the platform with specific credentials. The welcome screen will be the same for both groups and, similar to the envelopes, will not contain any information regarding the study sequence to ensure that participants are adequately blinded.

After clicking the start button, a first questionnaire designed to collect demographic data will be displayed (Multimedia Appendix 5). Learning preferences (visual, aural, reading/writing, or kinesthetic [VARK]) will be assessed using the online VARK questionnaire, which will be displayed in the student’s preferred idiom (either the French version translated by Johanne Barrette [29] or the German version translated by Greta Richter [30]), with permission from the original author (Heather Lander). This questionnaire consists of 16 multiple-choice items with four response options; possible scores range from 0 to 16 for each subscale. The VARK questionnaire has shown adequate validity and reliability [31]. Participants will access the VARK questionnaire by clicking on a link displayed on the online platform at the end of the first questionnaire; the results will be reported on a paper case report form (CRF; Multimedia Appendix 6) by one of the investigators before being entered directly on the study platform; a double check by the participant countersigning the document will be used to limit potential copy errors.

Both groups will then follow the e-learning module, the development of which has previously been described [32]. This module was initially developed in French and will be translated in German for the purpose of the study.

After completing the module, participants belonging to the control group will be asked to don the following PPE: protective glasses, FFP2 mask, coverall with hood, and gloves. They will then be asked to perform the doffing sequence individually, which will be recorded on video. After completing the doffing sequence, participants will be asked to go back to the online platform to electronically rebuild the doffing sequence.

Rather than immediately donning and doffing the PPE after completing the e-learning module, participants in the experimental group will be randomly allocated to the instructors to follow face-to-face learning according to the Peyton 4-step approach. After completing this workshop, these participants will resume the same path as their counterparts from the control group by moving on to the video recording of the doffing sequence and, finally, by being asked to rebuild the doffing sequence on the online platform.

Four to eight weeks later, depending on the schools’ schedules, participants will be asked to take part in a second session. This time interval is sufficient to reliably assess retention, which has been shown to be nonlinear. Out of the proportion of participants who display a significant decline in knowledge retention at 3 months, half will already present a significant decline in knowledge retention after 4 weeks [33]. Both groups will first repeat the video recording of the PPE doffing sequence and then reconnect to the platform to reconstruct the doffing sequence by computer. The experimental group will then be considered as having completed the study path, while the control group will attend a face-to-face learning session (See SPIRIT diagram in Multimedia Appendix 7 and Figure 1).

### Face-to-Face Learning

Face-to-face teaching will proceed according to the following steps based on the Peyton approach [14]: (1) the instructor will perform a complete doffing sequence in real time without any comments; (2) the instructor will perform a doffing sequence accompanied by step-by-step explanations (description of key points); (3) the learners will be asked to guide the instructor through the doffing sequence, step by step; (4) the learners will be asked to perform the complete doffing sequence before receiving individualized feedback. Each participant will perform this step only once.

Checklists and “buddy” systems have been used to improve the efficiency and security of doffing sequences [34,35]. Even though such techniques are undoubtedly useful, prehospital providers are required to perform PPE doffing procedures in many different locations, and sometimes lack access to either a “buddy” or even to a checklist. We therefore elected to refrain from giving any support document to the participants.

### Primary Outcome

The primary outcome will be the proportion of doffing sequences correctly performed after knowledge acquisition. Since practices differ from one center to another, we have developed an assessment grid validated by an IPC specialist for the assessment of this outcome (Multimedia Appendix 8). The adequacy of the procedure will be blindly assessed individually by two investigators (a prehospital emergency medicine expert and an IPC specialist) viewing the videos recorded in three-quarter view and using the developed checklist. In case of disagreement, a consensus will be reached by discussion.

### Secondary Outcomes

Seven secondary outcomes will be assessed: time required to teach the technique, time required to perform the doffing procedure, learner satisfaction, proportion of correct computer sequences, confidence in using PPE, and knowledge and skill retention.

The time required to teach the technique using the Peyton approach will be recorded on a paper CRF (Multimedia Appendix 9) by the instructor. Time will be measured from the beginning of step 1 until the last participant has completed the fourth and final step.

The time required to perform the doffing sequence will be recorded (in seconds) by analyzing the recording (measured from the moment the first item is taken off until the moment the learner announces that the procedure has been completed).

Learner satisfaction will be measured using a 5-point Likert scale (not satisfied at all, not satisfied, neutral/undetermined, satisfied, very satisfied).

Computerized doffing sequence accuracy involves reordering the presented sequence in random order in two steps: first in the contaminated zone and then in the noncontaminated zone.

Confidence in the ability to use PPE will also be assessed using a 5-point Likert scale (not confident at all, not confident, neutral/undetermined, confident, very confident).

Knowledge retention will be assessed by reordering the computer sequence once again 4-8 weeks after the first acquisition intervention, whereas skill retention will be assessed in the same way as the primary outcome.

### Blinded Data Collection and Assessment

Some outcomes will be recorded electronically. This will allow for their assessment to be independent from subjective human evaluation. For all other outcomes, assessors will be blinded to participant allocation.

Electronic data will be recorded and securely stored in an encrypted MariaDB database (Version 5.5.5; MariaDB Foundation) hosted on a Swiss server.

At the end of the study, all electronic data will be extracted to a comma-separated value file by the only investigator who will have access to the dataset (L Suppan). No personal data (name, first name, date of birth, or IP address) will be collected.

### Data Curation and Availability

An investigator (LC) will assign specific codes to the videotaped sequences of each participant. These codes will be created by concatenating the identifier used by the participant to log into the platform and the session number. These codes will be the only information sent to the blinded assessors apart from the recordings. All paper CRFs, including completed video assessment grids, will be sent to the investigator in charge of randomization (MS) for the constitution of the database. All electronically recorded data will then be imported to create the final version of the database. All data that could allow a data analyst to identify the group allocation will be deleted. The groups will be renamed otherwise (groups “Plutello” and “Plutinson”) and the curated database will be sent in Stata (Statacorp LLC) .dta file format to L Stuby for formal analysis. All investigators will be able to access the curated and coded dataset. The database will be deposited on Mendeley Data [36]. The videotapes will be used only for study purposes and destroyed once the curated data file has been created.

### Sample Size

According to two previous studies [12,13], the percentage of correct doffing sequence compliance through the use of the e-learning module should be very low, as no participant was able to recreate the correct sequence on the online platform in either study. However, practical reality might be dissociated from the theoretical answers gathered on online platforms, making a control group necessary in this study. For the record, a recent observational study showed that 90% of the doffing sequences were incorrect [37].

We calculated that 46 participants would be needed to have a 90% chance of detecting, at the 5% significance level, an increase in the primary outcome from 10% in the control group to 50% in the experimental group; additional participants will be accepted as the training will be part of their curriculum.

### Statistical Analysis

Data analysis will be performed using Stata 15.1. Owing to the small sample size, only nonparametric tests will be used. Fisher exact test will be used for dichotomous variables and the Mann-Whitney *U* test will be used for continuous variables. The computerized doffing sequence accuracy will first be analyzed as a whole and then according to the respective doffing zones (contaminated and noncontaminated). This should help determine whether further teaching efforts should be concentrated on a particular part of the sequence. The Likert scales will be described graphically and then dichotomized for statistical analysis (satisfied versus not satisfied; confident versus not confident). The results will be described as a percentage with 95% CI for the proportions and according to the median (Q1-Q3) for the continuous variables. A *P* value <.05 will be considered significant. A subgroup analysis by working status (actively working in an ambulance service or not) will be carried out as an increased rate in adequate choice of PPE has been shown in this subgroup [12]. Missing data will be excluded. There will be no adjustment or imputation.

## Results

The study has been presented to our regional ethics committee (Req-2020-01340), which waived the need for further evaluation by issuing a declaration of “no objection” as such projects do not fall within the scope of the Swiss federal law on human research [38]. The study will be performed in accordance to the principles of the Declaration of Helsinki [39] and Good Clinical Practice guidelines [40]. A formal agreement has already been obtained from the schools’ headmasters.

Once published, there will be no further modification to the study protocol. There is therefore no need to plan for communication of protocol amendments. This protocol version is 1.0 (January 11, 2021).

The online platform was finalized on January 15, 2021 [26]. The platform was developed by L Suppan and will be thoroughly tested by three coauthors (L Stuby, LC, MS). The current version of the welcome screen, with detailed information for five aspects (learning objectives, right to refuse participation or to withdraw consent at any time, institutional affiliation, and contact information), is displayed in Figures 2 and 3.

Study sessions in Geneva were performed on January 25, 2021 for instructor formation, January 26, 2021 for the first session, and February 24, 2021 for the second session. Study sessions in Bern are scheduled for April 2021 for instructor formation and for the first sessions, and for June 2021 for the second sessions.

The results, whether positive or negative, will be submitted for publication. They will be reported according to the CONSORT-EHEALTH checklist [18]. Relevant elements from the CHERRIES guidelines [19] will also be incorporated in the report.

## Discussion

### Main Considerations

This study should help determine whether face-to-face training using the Peyton 4-step approach in addition to the e-learning module can improve the application of a doffing procedure created by IPC specialists. It should also determine whether this approach improves knowledge and skill retention.

Four learning preference modalities have been previously described [41]: visual, which includes the depiction of information in diagrams, maps, graphs, arrows, circles, hierarchies, and other devices, that people use to represent what could have been presented in words; aural/auditory, which describes a preference for information that is “heard or spoken”; reading/writing, which describes preferences for information displayed as words; and kinesthetic, which refers to the “perceptual preference related to the use of experience and practice (simulated or real).” Although the e-learning module alone might suit the learning preferences of two of the four categories (visual and reading/writing) and face-to-face training may preferentially suit the visual, auditory, and kinesthetic categories, the combination of an e-learning module with face-to-face learning should theoretically suit all four categories.

Concerning our target population, assessment with first-year students who are new to the field and who have not yet been exposed to PPE use will allow us to test our hypothesis on learners who are still naive regarding the doffing method. These students should not be prejudiced and should not have developed any particular habit, good or bad, regarding the use of PPE.

One of the strengths of our study is that the videotapes will be independently assessed by an IPC expert and a prehospital emergency medicine expert.

We chose to design this study to assess the impact of face-to-face learning added to the e-learning module. Another interesting design would be to assess the gain of adding the e-learning module to face-to-face learning with the control group following the face-to-face learning alone and to the experimental group following the face-to-face learning and e-learning module. Although we considered creating a third group to test this hypothesis, we finally decided on the use of two groups given the limited sample size.

### Limitations

Some limitations can already be anticipated. First, the specific population of the sample may limit the generalizability of the results.

Second, using third-year students as instructors allows us to blind instructors, but can limit the quality of teaching. Indeed, these students who will be trained as an instructor within the framework of the study have no expertise in either IPC or in teaching, and have no or little experience in their profession and as an instructor. When peer students or student tutors are used as teachers, the effectiveness of the Peyton teaching approach is less clear [15].

The aim is to teach the technique for the first time, and therefore it will not be integrated in a care simulation and will be performed in a classroom setting. Therefore, the environment will not be representative of the actual situations in which the participants will have to perform these actions. This implies that the mental load on the learners will be lower than when they will have to apply these techniques in the field or integrated into simulations. Therefore, it could be beneficial, after learning the procedure, to train for actual application during simulated care situations.

### Conclusion

This study should help to determine whether face-to-face training in addition to an e-learning module can improve the application of a complex procedure and enhance its retention.

## Appendix

Multimedia Appendix 1 SPIRIT checklist.

Multimedia Appendix 2 PPE doffing procedure for instructors.

Multimedia Appendix 3 Peyton approach stages reminder sheet for instructors.

Multimedia Appendix 4 Copy of email to participants.

Multimedia Appendix 5 Questionnaire 1: demographic data.

Multimedia Appendix 6 VARK paper case report form (CRF).

Multimedia Appendix 7 SPIRIT diagram. Timeline of enrollment, interventions, and assessments.

Multimedia Appendix 8 Assessment grid.

Multimedia Appendix 9 Instructors paper case report form (CRF).

## Abbreviations

CRF: case report form
e-learning: electronic learning
IPC: infection prevention and control
PPE: personal protective equipment
VARK: visual, aural, reading/writing, or kinesthetic
