# Supplementary material for: Impact of Face-to-Face Teaching in Addition to Electronic Learning on Personal Protective Equipment Doffing Proficiency in Student Paramedics: Protocol for a Randomized Controlled Trial
Source: JMIR Res Protoc. 2021 Apr 30;10(4):e26927. doi: 10.2196/26927 (PMC8122292; doi:10.2196/26927)
Supplement: Multimedia Appendix 2 [file resprot_v10i4e26927_app2.docx]

This is a Multimedia Appendix to a full manuscript published in the JMIR Research Protocols. For full copyright and citation information see <http://dx.doi.org/10.2196/26927>

**PPE doffing procedure for instructors**

| French original version | English translated version |
| --- | --- |
|  |  |
| **Procédure retrait EPI – À destination des formateurs·rices** | **PPE doffing procedure – For instructors** |
| 1. Rester dans la zone contaminée | 1. Stay in contaminated zone |
| 2. Retrait et élimination non contaminant des gants (poubelle) | 2. Non-contaminating removal and disposal of gloves (trash can) |
| 3. Friction hydroalcoolique des mains | 3. Hydroalcoholic hand rubbing |
| 4. Ouvrir la combinaison | 4. Open the coverall |
| 5. Friction hydroalcoolique des mains | 5. Hydroalcoholic hand rubbing |
| 6. Enlever la capuche | 6. Take off the hood |
| 7. Ôter la combinaison en l’enroulant depuis l’intérieur, du haut jusqu’aux chevilles | 7. Take off the coverall by rolling it up from the inside, from the top to the ankles |
| 8. Jeter la combinaison dans une poubelle fermée | 8. Dispose the coverall in a closed trash can. |
| 9. Passer en zone « non-contaminée » | 9. Go in non-contaminated zone |
| 10. Friction hydroalcoolique des mains | 10. Hydroalcoholic hand rubbing |
| 11. Retrait non contaminant des protections oculaires | 11. Non-contaminating removal of eye protection |
| 12. Placer les lunettes dans un sachet, sans toucher celui-ci, pour désinfection ultérieure | 12. Place the glasses in a bag, without touching it, for subsequent disinfection |
| 13. Friction hydroalcoolique des mains | 13. Hydroalcoholic hand rubbing |
