# Supplementary material for: Impact of Face-to-Face Teaching in Addition to Electronic Learning on Personal Protective Equipment Doffing Proficiency in Student Paramedics: Protocol for a Randomized Controlled Trial
Source: JMIR Res Protoc. 2021 Apr 30;10(4):e26927. doi: 10.2196/26927 (PMC8122292; doi:10.2196/26927)
Supplement: Multimedia Appendix 3 [file resprot_v10i4e26927_app3.docx]

This is a Multimedia Appendix to a full manuscript published in the JMIR Research Protocols. For full copyright and citation information see <http://dx.doi.org/10.2196/26927>

**Peyton approach stages reminder sheet for instructors**

| French original version | English translated version |
| --- | --- |
|  |  |
| 1) Le formateur réalise une séquence de déshabillage complète en temps réel | 1) The instructor performs a complete doffing sequence in real time |
| 2) Le formateur réalise une séquence de déshabillage avec les explications étape par étape (description des points-clés) | 2) The instructor performs a doffing sequence with step-by-step explanations (description of key points) |
| 3) Les apprenants guident étape par étape le formateur qui effectue le déshabillage | 3) The learners guide the instructor step by step to perform the doffing |
| 4) Les apprenants effectuent le déshabillage en réalisant la séquence complète et lorsque celle-ci est terminée, le formateur lui fait un retour. Chaque participant n’effectue cette étape qu’une seule fois. Aucun support (par exemple ordinateur, tableau blanc, checklist) ne sera utilisé pour rappeler à l'étudiant les étapes de la procédure | 4) The learners do the complete doffing sequence then a feedback is given from the instructor. Each participant performs this step only once. No support (i.e. computer, whiteboard, checklist) will be used to remind the student the steps of the procedure |
