# Supplementary material for: Impact of Face-to-Face Teaching in Addition to Electronic Learning on Personal Protective Equipment Doffing Proficiency in Student Paramedics: Protocol for a Randomized Controlled Trial
Source: JMIR Res Protoc. 2021 Apr 30;10(4):e26927. doi: 10.2196/26927 (PMC8122292; doi:10.2196/26927)
Supplement: Multimedia Appendix 4 [file resprot_v10i4e26927_app4.docx]

This is a Multimedia Appendix to a full manuscript published in the JMIR Research Protocols. For full copyright and citation information see <http://dx.doi.org/10.2196/26927>

**English translation of copy of email to participants**

*Dear first-year student,*

*During your coming practical workshop’s days, you will follow a training about the effective use of personal protective equipment (PPE) used during the care of COVID’s patients. This training will be provided to you in the frame of my graduate’s thesis, to prepare you as well as possible to your futures traineeships. One of the 4 workshops planned during these days will be dedicate to this practical training, either on first or second session and will be provided by a third’s years paramedic student. Furthermore, you will be video-recorded while you practice the procedure: these videos are only used in an investigational purpose, they won’t be spread and will be deleted thereafter. More information about this will be given to you during the first session.*

*During these two days, you must attend with your complete uniform (including protective glasses and safety shoes). A part of the training will be on an informatic support, so you will be asked to also bring your computer or smartphone during these two sessions.*

*Kind regards,*

*On behalf the investigators,*

*Ludivine Currat*
