# Supplementary material for: Impact of Face-to-Face Teaching in Addition to Electronic Learning on Personal Protective Equipment Doffing Proficiency in Student Paramedics: Protocol for a Randomized Controlled Trial
Source: JMIR Res Protoc. 2021 Apr 30;10(4):e26927. doi: 10.2196/26927 (PMC8122292; doi:10.2196/26927)
Supplement: Multimedia Appendix 5 [file resprot_v10i4e26927_app5.docx]

This is a Multimedia Appendix to a full manuscript published in the JMIR Research Protocols. For full copyright and citation information see <http://dx.doi.org/10.2196/26927>

**Questionnaire 1 demographic data**

| French original version | English translated version |
| --- | --- |
|  |  |
| Vous êtes :  - Un homme  - Une femme  - Autre | You are:  - A man  - A woman  - Other |
| Quel est votre âge ? | How old are you? |
| Vous êtes étudiant·e à :  - Genève (ESAMB)  - Lausanne (ES-ASUR)  - Berne – francophone (MEDI)  - Berne – germanophone (MEDI) | You are student in:  - Geneva (ESAMB)  - Lausanne (ES-ASUR)  - Bern – French-speaking (MEDI)  - Bern – German-speaking (MEDI) |
| Travaillez-vous dans un service d’ambulance en parallèle de vos études ?  - Oui  - Non | Do you actively work in an ambulance service during your studies?  - Yes  - No |
| Si oui, dans quel canton ?  - Vaud  - Neuchâtel  - Fribourg  - Berne  - Genève  - Valais  - Jura  - Autre | If yes, in which canton?  - Vaud  - Neuchâtel  - Fribourg  - Bern  - Geneva  - Valais  - Jura  - Other |
