# Supplementary material for: Impact of Face-to-Face Teaching in Addition to Electronic Learning on Personal Protective Equipment Doffing Proficiency in Student Paramedics: Protocol for a Randomized Controlled Trial
Source: JMIR Res Protoc. 2021 Apr 30;10(4):e26927. doi: 10.2196/26927 (PMC8122292; doi:10.2196/26927)
Supplement: Multimedia Appendix 6 [file resprot_v10i4e26927_app6.docx]

This is a Multimedia Appendix to a full manuscript published in the JMIR Research Protocols. For full copyright and citation information see <http://dx.doi.org/10.2196/26927>

**VARK paper CRF**

| **Username** | **V** | **A** | **R** | **K** | **Investigator signature** | **Participant signature** |
| --- | --- | --- | --- | --- | --- | --- |
|  |  |  |  |  |  |  |
|  |  |  |  |  |  |  |
|  |  |  |  |  |  |  |
|  |  |  |  |  |  |  |
|  |  |  |  |  |  |  |
|  |  |  |  |  |  |  |
|  |  |  |  |  |  |  |
|  |  |  |  |  |  |  |
|  |  |  |  |  |  |  |
|  |  |  |  |  |  |  |
|  |  |  |  |  |  |  |
|  |  |  |  |  |  |  |
|  |  |  |  |  |  |  |
|  |  |  |  |  |  |  |
|  |  |  |  |  |  |  |
|  |  |  |  |  |  |  |
|  |  |  |  |  |  |  |
|  |  |  |  |  |  |  |
|  |  |  |  |  |  |  |
