# Supplementary material for: Impact of Face-to-Face Teaching in Addition to Electronic Learning on Personal Protective Equipment Doffing Proficiency in Student Paramedics: Protocol for a Randomized Controlled Trial
Source: JMIR Res Protoc. 2021 Apr 30;10(4):e26927. doi: 10.2196/26927 (PMC8122292; doi:10.2196/26927)
Supplement: Multimedia Appendix 7 [file resprot_v10i4e26927_app7.docx]

This is a Multimedia Appendix to a full manuscript published in the JMIR Research Protocols. For full copyright and citation information see <http://dx.doi.org/10.2196/26927>

|  | **Enrollment** | **Allocation** | **Post-allocation session**  **(Acquisition)** | | **Follow-up session**  **(Retention)** |
| --- | --- | --- | --- | --- | --- |
| **TIMEPOINT** | ***- 10 days*** | ***t_0_*** | ***+ 10 minutes*** | ***+ 45 minutes*** | ***+ 4-6 weeks*** |
| **ENROLMENT:** |  |  |  |  |  |
| **Email invitation** | X |  |  |  |  |
| **Informed consent** |  | X |  |  |  |
| **Envelope choosing (allocation)** |  | X |  |  |  |
| **INTERVENTIONS:** |  |  |  |  |  |
| **Control group**  ***[E-learning]*** |  |  | X |  |  |
| ***[Face-to-face workshop]*** |  |  |  |  | X |
| **Experimental group**  ***[E-learning]*** |  |  | X |  |  |
| ***[Face-to-face workshop]*** |  |  | X |  |  |
| **ASSESSMENTS:** |  |  |  |  |  |
| ***Demographic data*** |  | X |  |  |  |
| ***Learning preferences (VARK)*** |  | X |  |  |  |
| ***Teaching time*** |  |  | X |  |  |
| ***Computer sequence*** |  |  |  | X | X |
| ***Satisfaction*** |  |  |  | X |  |
| ***Confidence in using PPE*** |  |  |  | X |  |
| ***Video recording of sequences*** |  |  |  | X | X |

**SPIRIT Figure. Timeline of enrolment, interventions and assessments.**
