# Supplementary material for: Impact of Face-to-Face Teaching in Addition to Electronic Learning on Personal Protective Equipment Doffing Proficiency in Student Paramedics: Protocol for a Randomized Controlled Trial
Source: JMIR Res Protoc. 2021 Apr 30;10(4):e26927. doi: 10.2196/26927 (PMC8122292; doi:10.2196/26927)
Supplement: Multimedia Appendix 8 [file resprot_v10i4e26927_app8.docx]

This is a Multimedia Appendix to a full manuscript published in the JMIR Research Protocols. For full copyright and citation information see <http://dx.doi.org/10.2196/26927>

**Assessment grid**

| French original version | English translated version |
| --- | --- |
|  |  |
| **Numéro de la session (1 ou 2)** | **Session number (1 or 2)** |
| **Identifiant du participant** | **Participant identifier** |
| **Durée de la séquence** *(en secondes ; dès que le premier élément est saisi jusqu’à ce que la procédure soit signalée comme terminée)* | **Sequence duration** *(in seconds; from when the first element is taken until the procedure is completed)* |
|  | |
| **Déshabillage**  *Les étapes suivantes doivent être suivies dans l’ordre. L’absence d’une des actions citées ci-après OU le non-respect de l’ordre de la séquence représente une contamination (échec de la procédure)* | **Doffing**  *The following steps should be taken in order. The absence of one of the actions listed below OR the non-compliance with the order of the sequence represents contamination (failure of the procedure*) |
| **En zone contaminée** | **In contaminated zone** |
| □ Retrait non contaminant des gants | □ Non-contaminating glove removal |
| □ Friction hydroalcoolique des mains | □ Hydroalcoholic hand rubbing |
| □ Ouverture de la combinaison | □ Coverall opening |
| □ Friction hydroalcoolique des mains | □ Hydroalcoholic hand rubbing |
| □ Retrait de la combinaison sans contact avec l’extérieur de celle-ci (prêter particulièrement attention à la capuche) ; la jeter dans une poubelle fermée | □ Coverall removal without outside contact of it (pay particular attention to the hood); throw it in a closed trash can |
| **En zone non-contaminée** | **In non-contaminated zone** |
| □ Friction hydroalcoolique des mains | □ Hydroalcoholic hand rubbing |
| □ Retrait des lunettes de protection ; les placer dans un sachet (sans le toucher) pour une désinfection ultérieure | □ Protective glasses removal; placed them in a bag (without touching it) for subsequent disinfection |
| □ Friction hydroalcoolique des mains | □ Hydroalcoholic hand rubbing |
