# Supplementary material for: Impact of Face-to-Face Teaching in Addition to Electronic Learning on Personal Protective Equipment Doffing Proficiency in Student Paramedics: Protocol for a Randomized Controlled Trial
Source: JMIR Res Protoc. 2021 Apr 30;10(4):e26927. doi: 10.2196/26927 (PMC8122292; doi:10.2196/26927)
Supplement: Multimedia Appendix 9 [file resprot_v10i4e26927_app9.docx]

This is a Multimedia Appendix to a full manuscript published in the JMIR Research Protocols. For full copyright and citation information see <http://dx.doi.org/10.2196/26927>

**Instructors’ paper CRF**

| French original version | English translated version |
| --- | --- |
|  |  |
| **Date** | **Date** |
| **Nom du formateur** | **Instructor’s name** |
| **Nombre d’apprenants** | **Number of learners** |
| **Heure du début de la formation** *(format HH:MM ; début de l’étape 1)* | **Start time of workshop** *(HH:MM format; from start of step 1)* |
| **Heure de fin de la formation** *(format HH:MM ; fin de l’étape 4 pour le dernier participant)* | **End of training time** *(HH:MM format; to the end of step 4 for the last participant)* |
